# Biocomposites and Poly(lactic acid) in Active Packaging: A Review of Current Research and Future Directions

**DOI:** 10.3390/polym17010003

**Published:** 2024-12-24

**Authors:** Sofiane Akhrib, Souad Djellali, Nacereddine Haddaoui, Davud Karimian, Mauro Carraro

**Affiliations:** 1 Laboratory of Physical-Chemistry of High Polymers, Faculty of Technology, University Ferhat Abbas—Setif 1, Setif 19000, Algeria; 2 Department of Chemistry, Faculty of Sciences, University Ferhat Abbas—Setif 1, Setif 19000, Algeria; 3 Department of Chemical Sciences, University of Padova, Via Marzolo 1, 35131 Padova, Italy; 4 Institute on Membrane Technology (ITM-CNR), UOS of Padova, Via Marzolo 1, 35131 Padova, Italy

**Keywords:** biocomposites, poly(lactic acid), active packaging, smart polymer, biodegradable, renewable polymer

## Abstract

The alarming rise in environmental pollution, depletion of global resources, and increasing health consciousness have placed significant pressure on the development of eco-friendly, sustainable materials. Consequently, green, environmentally friendly materials made from biobased and/or biodegradable sources are gaining recognition and political support as sustainable alternatives to petroleum-based, non-biodegradable materials. Bio-based packaging materials, in particular, are widely used across all industrial sectors, with a growing demand for solutions that preserve food quality and extend shelf life. Within this context, the concept of “active packaging” (AP) is attracting considerable interest. While the traditional view of packaging materials is that they should be basically inert, active packaging involves intentional interactions with the packaged product or surrounding atmosphere, providing enhanced protection against degradation caused by human actions and environmental factors. This work aims to highlight the significant impact of biocomposites in the active packaging sector, driven by the synergistic integration of nanofillers and active agents, while providing an in-depth analysis of the key mechanisms and strategies underlying their functionality. Particular emphasis is placed on poly(lactic acid)(PLA), presenting a comprehensive review of innovative approaches to enhance the performance of PLA-based packaging, with a focus on improving antioxidant and antimicrobial properties to meet the demands of sustainable and efficient packaging solutions.

## 1. Introduction

Plastics, though indispensable to modern technological progress, present profound challenges that overshadow their benefits. The term “Plastic Age” not only highlights their ubiquity but also underscores the environmental crisis they contribute to [1,2]. This has spurred a growing demand for eco-friendly alternatives [3], driving significant research into bio-based polymers featuring high performance and cost efficiency. As a result, several biopolymers and biocomposites derived from renewable sources are being developed and tailored to meet sustainability goals [4,5,6,7,8,9]. For instance, starch-based materials are now engineered into plastics suitable for a wide range of applications [10].

**Figure 1:**
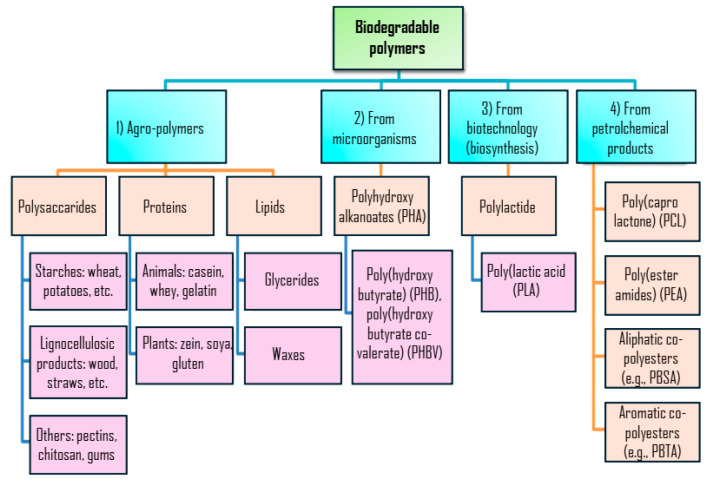
Classification of biodegradable polymers.

In this context, biodegradable plastic production was estimated to be 1.136 million tons in 2023, representing less than 0.3% of the current annual production of plastics [11]. Biodegradable plastics are designed to decompose through microbial action into water, carbon dioxide (or methane), and biomass under specific environmental conditions, (e.g., temperature, humidity, and microbial activity) [12]. These plastics fall into four distinct families [13]: (i) agro-polymers derived from biomass products such as starch, cellulose, proteins, and chitin; (ii) biopolymers produced via microbial metabolism, such as polyhydroxyalkanoates (PHA); (iii) biopolymers where the monomers are sourced from biological materials, such as PLA, produced through biotechnological processes; and (vi) conventional chemically synthesized polymers [14,15]. This classification is summarized in Figure 1.

**Figure 2:**
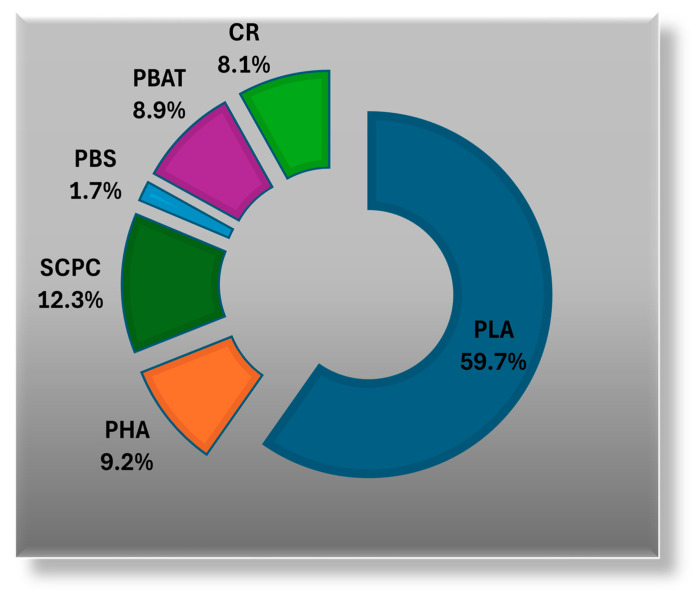
The global production capacities of biodegradable plastics in 2023. Adapted with permission from ref. [16].

Figure 2 shows the most recent data from “European Bioplastics” in collaboration with the novice institute (Hürth, Germany), where polylactic acid (PLA) represents ~60% of the market share of biodegradable plastics [16].

PLA is a widely used commercial biodegradable polymer and an excellent alternative to fossil-based plastic products. It is a thermoplastic aliphatic polyester derived from agricultural crops such as rice, corn, and sugar beets [17], that has a wide variety of applications, e.g., in biomedicine [18,19], the automotive industry, and food packaging [20,21].

## 2. Biocomposites Development and Challenges

Composite materials are the result of combining two ingredients that are non-miscible: a continuous phase, known as the matrix, and the dispersed phase. The main concept is to gain properties that could not be obtained by using the ingredients alone [22]. A typical composite consists of a stronger reinforcement phase, often a fiber, embedded in a weaker, malleable matrix. The matrix transfers stress to the reinforcement while protecting it from mechanical and environmental damage, enabling the reinforcement’s strength to be effectively utilized [23].

A material is classified as a biocomposite when at least one of its components, such as the matrix or reinforcement, is biobased [24]. Biocomposites are gaining significant interest across various industries due to their excellent insulating properties (both electronic and thermal) and impressive wear resistance. These materials offer competitive advantages not only because of their renewable nature but also due to the low density, high aspect ratio, and reactive surfaces of many natural fibers. More-over, with growing environmental concerns and stricter regulations on conventional materials, biocomposites present a more sustainable and attractive long-term solution for diverse sectors [25,26,27,28,29]. Within this scenario, biocomposites, made from plant fibers and biodegradable plastics, are anticipated to play a crucial role in the composite material industry [3].

Given that packaging accounts for 41% of global plastic consumption—primarily in food packaging—there is growing interest in the use of biocomposites as a sustainable alternative in this domain [30]. However, many biocomposites remain in the research and development phase, with only a limited selection commercially available. These are often based on thermoplastic polymers reinforced with natural fibers such as flax, hemp, jute, or kenaf [31]. Consequently, significant efforts are focused on developing new processing and production technologies for making biocomposites appealing for the market [32].

A variety of methodologies have been adopted to prepare biocomposites, reflecting the industry’s need for flexible and innovative approaches. Nevertheless, the production of biocomposites still faces notable hurdles, particularly in transitioning biobased active packaging systems from laboratory research to industrial-scale applications. Among the primary scientific challenges is the limited mechanical strength of many biobased matrices, which reduces their stability under environmental conditions such as temperature and humidity. Poly(lactic acid) (PLA) stands out as a promising candidate due to its availability, cost-effectiveness, effective barrier properties, and low toxicity compared to alternatives like cellulose, gelatin, chitosan, and plant-based oils [33]. Moreover, ensuring compatibility between active agents, nanofillers, or reinforcement materials and biopolymer matrices remains complex. One critical issue is the incompatibility between hydrophobic polymer matrices and hydrophilic fibers, resulting in poor interfacial adhesion, uneven fiber distribution, and diminished mechanical performance [34]. Addressing this requires significant advancements in functionalization strategies to achieve uniform dispersion and robust bonding. Additionally, sustained activity and controlled release of active agents throughout the shelf life of packaged foods demand optimization through intelligent release strategies [35].

A comprehensive understanding of biocomposites’ interactions with diverse food matrices and detailed life cycle assessments (LCAs) to evaluate environmental impacts are also critical areas of ongoing research [36].

Beyond scientific challenges, external barriers hinder the adoption of biobased active packaging in the food industry. High production costs and scalability issues make biocomposites less competitive than conventional plastics. Packaging typically accounts for around 10% of a product’s total cost, but incorporating active packaging can double this expense. This increase in cost presents challenges to the affordability of active packaging, making it less attractive for widespread adoption in the industry. Moreover, existing industrial packaging machinery may not be compatible with biobased materials, adding to manufacturing difficulties. As a matter of fact, techniques commonly used in polymer processing, such as extrusion, pultrusion followed by injection molding, compression molding, solvent or solution casting, and in situ intercalative polymerization, should be adapted for biocomposites [28,37].

The lack of clear regulatory frameworks and the need for different certification systems poses significant barriers to the market entry of active packaging. Limited consumer awareness and skepticism about the safety and benefits of biobased packaging further hinder adoption. For example, evaluating active materials based on exposure levels or toxicological profiles can significantly influence production processes. Additionally, the potential allergenicity of certain essential oils used in active packaging highlights the importance of compatibility considerations. Challenges are further compounded by concerns over aesthetic and sensory properties, such as transparency, texture, and odor, which can impact consumer acceptance.

Lastly, the underdeveloped supply chain for biobased raw materials and resistance from markets accustomed to conventional packaging underscore the need for interdisciplinary collaboration among researchers, industry stakeholders, and policymakers to overcome these obstacles and drive practical applications [38].

## 3. Relevance of Active Packaging

In active packaging, the goal is to fulfill a specific or desired function in preserving the product beyond merely acting as a passive barrier against external factors and improving the performance of the packaging system [39]. According to European Regulation (EC) No 450/2009, an active packaging system interacts with food by intentionally including components that release or absorb substances into or from packaged food or its surrounding environment (European Commission, 2009) [40]. One of the major advantages of this technology is that it enables one or more strategies at once, depending on the characteristics or attributes that need to be preserved. For instance, if the product is being attacked by oxidation, an oxygen scavenger could simply be used, while if it is suffering from moisture or water vapor problems, a moisture absorber could be included [41].

**Figure 3:**
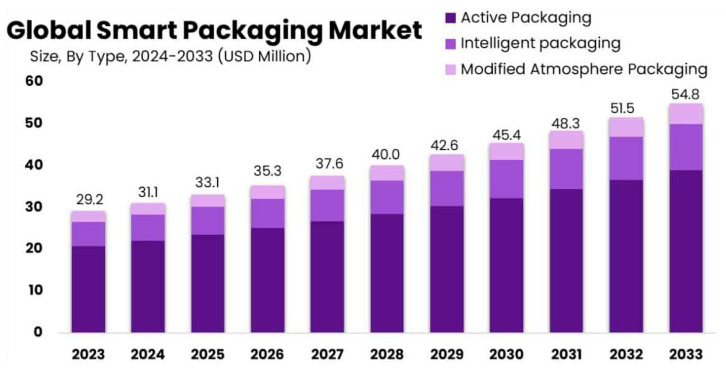
Expected global trend of active packaging market. Reproduced with permission from Market.us (ref. [42]).

The global smart packaging market size is projected to expand significantly by 2033, with a compound annual growth rate (CAGR) of approximately 6.5% [42]. Besides the prominent role of active packaging (AP), other innovative technologies, such as intelligent packaging [43] and modified atmosphere packaging, are also experiencing rapid growth and contributing to the market’s evolution (Figure 3). While active packaging has diverse applications across multiple industries, our focus will center on its role in food-related applications, where it is essential for preserving freshness, extending shelf life, and enhancing the overall quality and safety of food products.

### 3.1. Active Packaging Systems for Food

In an active packaging system, the packaging material comes in contact with the product to help protect it: the critical factor in this technology is the understanding of the specific sensitivities of the product, in order to devise the most effective countermeasures [44].

**Figure 4:**
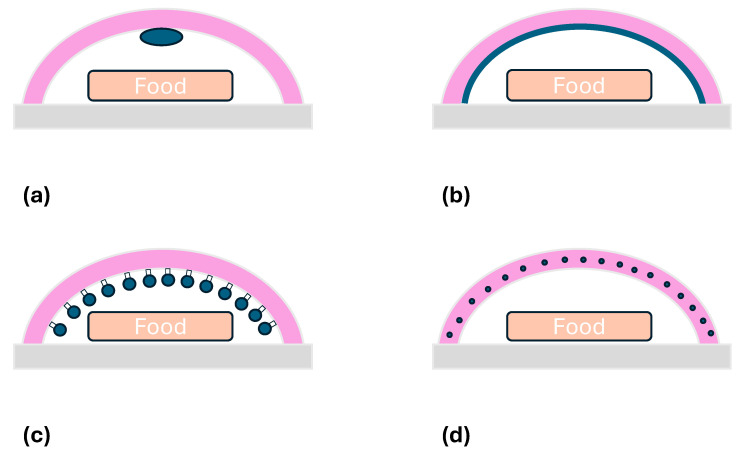
The common forms of AP systems including active agents: (**a**) sachets; (**b**) coating or adsorption; (**c**) immobilization via ionic or covalent bonds; (**d**) embedding in the matrix.

The AP systems for food packaging [41] can be designed in various ways, as illustrated in Figure 4: addition of sachets or pads containing active agents; coating or adsorption of active compounds onto the polymer surface; immobilization of active compounds on polymers via ionic or covalent bonds; and incorporation of active compounds in the polymer matrix.

#### 3.1.1. Active Packaging: Mechanisms of Action

**Figure 5:**
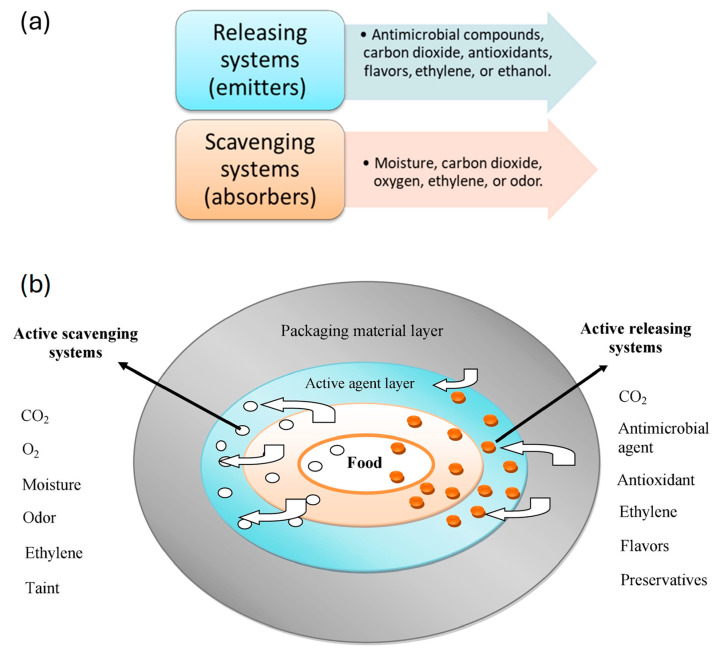
(**a**) Active packing classification based on the mechanism of action; (**b**) examples of active (scavenging/releasing) systems used in the meat industry (reproduced with permission from ref. [44]; copyright 2017 Elsevier B.V.).

Based on the mechanism of action, active packaging systems can be classified into “releasing systems” and “absorbing systems” (Figure 5). As their name implies, an emitting or releasing system liberates the active agent into the space around the food or its surface. This enables the agent to perform its specific function, such as preserving quality or imparting desired characteristics to the package. As a matter of fact, an absorbing or scavenging system captures undesirable substances that may accumulate over time, thereby also helping to preserve the quality of the product for as long as possible.

##### Release Control

In general, when the active agent is uniformly incorporated into the polymer matrix (Figure 4d), its release is usually triggered in one of the following ways [45]:

- By diffusion: in porous polymers, the active agent migrates, through the matrix, from the film towards the food;
- By swelling: unlike in the first type, in this method, the agent is unable to slip through the polymer; however, the latter may swell when placed in compatible liquids, creating the conditions for the agent to be released;
- Disintegration: this method takes advantage of the deformation or degradation that may occur in the packaging material over time, allowing the agent stored in the matrix to be subsequently released.

One of the greatest concerns in an AP system is related to the compound migration rate. Due to their low molecular weight, indeed, most agents have fast release rates, strongly affecting the possibility to achieve a prolonged action of the AP. Therefore, an important consideration in packaging is to devise a system to maintain the agent for longer periods of time, ultimately leading to a notable increase in shelf life [41].

Various strategies are employed to enhance the properties of packaging materials, including chemical modification of polymers, irradiation treatment, cross-linking, and laminated film fabrication [46]. Additional methods focus on altering the properties of active compounds to reduce their volatility or control their diffusion, such as micro- or nanoencapsulation. When incorporated in the form of nanoparticles, these can serve as reservoirs for active compounds, enabling precise control over the timing and rate of their release. Furthermore, nanoparticles may also act as nanoreinforcements, significantly improving the mechanical strength, barrier performance, and thermal stability of the packaging film [45].

#### 3.1.2. Types of Active Packaging System

Many tasks have been developed for active packaging in general [40], and they are summarized as follows:

- Antioxidants and oxygen scavenging;
- Carbon dioxide emitting–absorbing systems;
- Ethylene scavengers;
- Flavor and odor releasers/absorbers;
- Antimicrobials;
- Moisture absorbers.

**Figure 6:**
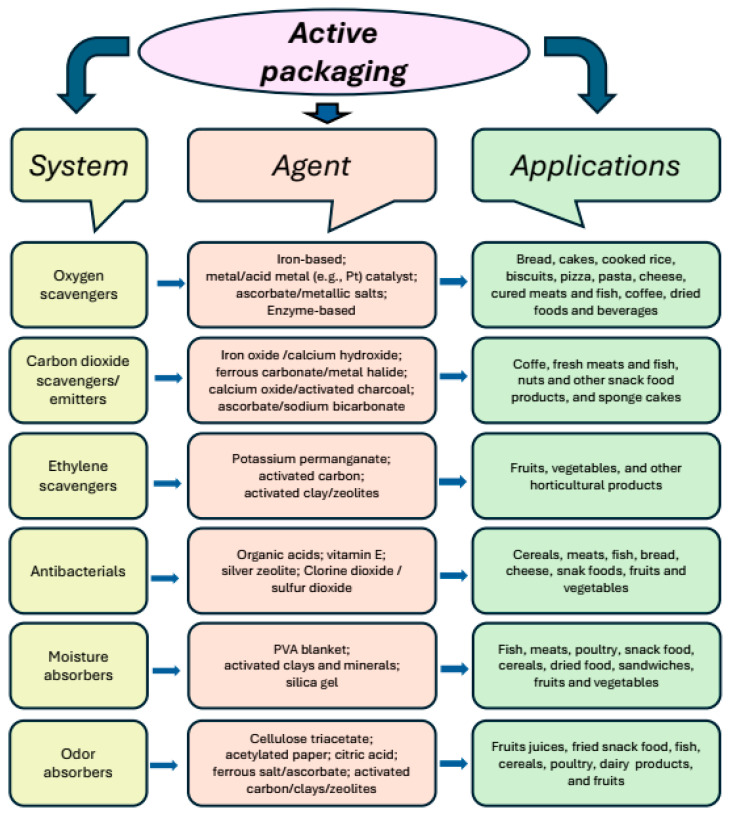
Some active packaging systems and their applications in the food industry.

Figure 6 provides a general overview of active packaging systems. The specific system are shown on the left, examples of agents used are in the center, and some applications are listed on the right.

##### Oxygen Scavengers and Antioxidants

Oxygen is one of the most critical factors contributing to food spoilage. Residual oxygen present during the packaging process can react with food components, triggering biochemical and chemical reactions that lead to quality degradation and product deterioration [47]. The classic method of counteracting the effects of oxygen is by removing it through vacuum sealing, inert gas flushing (using N_2_ and CO_2_), or a combination of both. This generally helps eliminate 90 to 95% of the oxygen inside the package [48].

Alternatively, oxygen scavenger iron-based sachets can be used to reduce residual oxygen to levels as low as 0.01%, significantly outperforming modified atmosphere packaging. This effectiveness is crucial for protecting oxygen-sensitive products, such as vacuum-sealed meats, ensuring optimal preservation. Two major advantages can be achieved [49]:

- Inhibition of oxidation: oxygen scavengers work to effectively prevent oxidation processes by incorporating O_2_ into oxidized salts or molecules. For example, in the packaging of snacks and coffee, iron powder-based oxygen scavengers remove oxygen from the sealed environment, thereby slowing down food degradation and maintaining product freshness.
- Microbial control: oxygen scavengers are used to inhibit the growth of aerobic microorganisms, including bacteria and molds. This is particularly important for extending the shelf life of bakery products or dried fruits.

On the other hand, to mitigate concerns about potential metallic taints in food products, nonmetallic oxygen scavengers have been developed. These agents include organic reducing agents such as ascorbate salts, ascorbic acid (vitamin C), or catechol; additionally, enzymatic oxygen scavenger systems utilizing either glucose oxidase or ethanol oxidase have been utilized [50].

However, oxygen can sometimes infiltrate hermetically sealed packages due to various reasons and initiate oxidation processes involving reactive oxygen species (ROS) such as oxo-, hydroxyl-, and superoxide radicals. Therefore, eliminating these radicals can prevent extensive oxidation. Antioxidant agents have thus gained substantial popularity in packaging applications due to their ability to extend the shelf life of perishable products [38]. Tongdeesoontorn et al. developed biocomposite films made by cassava starch/gelatin, enriched with quercetin and tert-butylhydroquinone (TBHQ). These films demonstrated the ability to retard oxidation processes in food, particularly in meat products [51]. In another study, vegetable waste served as a renewable source of antioxidants, which were integrated into a matrix of carbon dioxide-derived poly(propylene carbonate) (PPC) polymer, bringing an effective antioxidant activity to the packaging film [52].

##### Carbon Dioxide Emitting or Scavenging Systems

Carbon dioxide (CO_2_) is used as a preservative for certain food products, primarily for its ability to inhibit the growth of aerobic bacteria and molds, which require oxygen. This method is common in the modified atmosphere packaging (MAP) of fresh meat, cheese, fish, and other perishables. A CO_2_ environment inhibits bacterial growth, extending the shelf life of these products [41]. Freshly roasted or ground coffee emits significant amounts of CO_2_, which can cause bloating in sealed coffee packages. Activated charcoal and calcium hydroxide are used in packaging to absorb excess CO_2_, preventing package deformation and preserving product quality [53]. Besides CO_2_ emitting or scavenging systems, the CO_2_ gas permeability of the packaging layer is also crucial to balance its amount.

In a recent study, a biodegradable film of poly(butylene adipate-co-terephthalate) (PBAT) and thermoplastic starch (TPS) was synthesized and 3–5% of ZnO was incorporated in it. This biocomposite has shown an effect on the antimicrobial activity, also modulating water and gas transport properties. The authors have reported that dispersing ZnO into the PBAT/TPS film allowed for a balance between water absorption and CO_2_ emission, achieving a 35% balance in CO_2_ emission [54]. Polysaccharides, like starch, chitosan, and cellulose, are often used in biodegradable packaging due to their good barrier properties for gases. Their ability to limit CO_2_ and O_2_ permeability, indeed, makes them effective for food packaging applications [55].

##### Ethylene Scavengers

When fruits and vegetables are ready to harvest, they start to produce ethylene, which is a phytohormone that initiates and accelerates the ripening process; this simply means that ethylene scavenging plays a major role in the storage, processing and shelf life of fruits and vegetables. To this aim, suitable agents are oxides (i.e., silica gel), aluminosilicate minerals (e.g., clays, vermiculite, and zeolite), and activated carbon [40,41]. For example, Kumar et al. reported the incorporation of clays into different types of polymers, including starch-based films, for ethylene removal in fresh-produced products. They mentioned that some factors like clay-specific surface area, pore volume, storage temperature, and humidity significantly affect the ethylene removal capacity [56]. In another study, flexible films made of cellulose nanofibers doped with Cu_2_O-modified TiO_2_ nanotubes were synthesized. These films exhibited strong antibacterial activity and were used in tomato packaging to assess their ethylene scavenging properties [57].

##### Antibacterials

The greatest cause of food spoilage is bacterial activity; many species are known, such as *Salmonella* spp., *Bacillus cereus*, *Aspergillus* (molds), and *Candida* (yeasts) [41]. The traditional approach to addressing this issue includes several methods, such as heat treatment, freezing, and modified atmosphere packaging. However, active packaging includes the antibacterial agents in the matrix or on the surface of the packaging material [40]: the most common, commercially available agents are silver-based materials such as Biomaster^®^, Aglon^®^, and Bactiblock^®^ [48].

Srivastava et al. reported a biocomposite based on corn starch, reinforced by rice husk fibers and including benzalkonium chloride as an antimicrobial agent. The authors showed that a variation in the content of components led to good antimicrobial properties against food-spoilage bacteria [58]. In another study, the usage of essential oils (EOs) embedded into starch-based films was shown to have a good influence on the prevention of food deterioration by bacteria and fungi [59]. Diken et al. prepared a biocomposite based on polycaprolactone (PCL) with ferulic and gallic acid through a melt blending method. This biocomposite not only demonstrated higher storage modulus but also exhibited high-scavenging activity against organic radicals and high-antibacterial activity against *Escherichia coli* and *Staphylococcus aureus* bacteria [60].

##### Moisture Control

Foods such as meat and vegetables often accumulate excess water within their packaging over time, promotes food spoilage, increases bacterial activity, and alters texture and appearance. In these cases, using a moisture absorber effectively mitigates quality degradation and preserves taste, keeping the food fresh for a longer period. Moisture control technologies currently dominate the market compared to other active packaging solutions. Common forms include liquid absorbers, such as pads and sheets, and humidity regulators available as sheets or tags [61]. Some highly resistant substances could also be incorporated into plastic films to create desiccant structures [62]. It was reported, for example, that making biocomposites including carboxymethyl cellulose, obtained from coconut coir and groundnut shell, along with olive oil, lead to good water barrier properties in food packaging [63]. In another study, biocomposite film, obtained using whey protein isolate, polyvinyl alcohol, and colloidal nano-silica, showed good moisture control. The authors demonstrated that the addition of nano-silica as a reinforcement could improve tensile strength, leading to good water vapor barrier capability [64]. Biocomposite films produced by cellulose and alginate were modified by the addition of microfibrillated and nanofibrillated cellulose as reinforcement. This biocomposite structure could impart good barrier properties and reduce water vapor permeation [65].

##### Flavor and Odor Absorbers/Releasers

When consumers open a food package, the odor released can impact product desirability. For this reason, considerable effort has been invested into developing packaging materials that selectively eliminate unwanted odors or neutralize the compounds responsible for them. One example is the incorporation of vitamin E into packaging materials for snacks and baked goods to enhance the product’s appeal [40]. Accordingly, gelatin-based films containing microcrystalline cellulose and various oleoresins have been shown to improve food packaging by reducing water vapor permeability and preserving the taste and flavor quality of foods, especially bread [66]. In another study, encapsulation systems were investigated to control the release of active compounds that enhance packaging quality in terms of aroma and flavor. This approach is particularly beneficial for temperature- or oxygen-sensitive flavors, such as citrus oil, as it helps protect them from oxidative and thermal degradation during processing. For instance, the use of encapsulated citrus and cardamom flavors in candies and cakes, through molecular inclusion in cyclodextrin, has been reported to maintain flavor integrity [67].

#### 3.1.3. Smart Packaging

In addition to the task-specific active agents mentioned above, several intelligent or smart packaging systems incorporating sensors have been developed. These systems are primarily designed to monitor the product and provide valuable information about the package’s condition and history, significantly aiding in the prevention of food spoilage and enhancing logistics efficiency. Several indicators can be used: for example,

- Time temperature indices (TTIs) (based on chemical, physical, biological, or enzymatic processes) [68];
- Seal and break indicators (using CO_2_ and O_2_ indicators in general) [48];
- Freshness indicators that monitor volatile metabolite concentrations produced during food aging [40].
- Color sensors, used as labels to monitor food quality and provide safety information in case of issues during the packaging process. These sensors detect critical changes by tracking color shifts, which can indicate factors such as food freshness or the presence of microbial metabolites [69].

#### 3.1.4. Nanotechnology in Active Packaging

Studies have shown that incorporating nanotechnology into packaging systems can significantly enhance food quality, extend shelf life, and maintain food safety. Nano-sized materials and nanoreinforcements impact various properties of biocomposites [70]. AP systems incorporating nanoparticles, indeed, exhibit significantly improved barrier properties, including reduced permeability to oxygen, moisture, and other gases. Additionally, these nanocomposite materials enhance thermal properties such as increased melting points and glass transition temperatures, ensuring better performance under various environmental conditions. Mechanical behaviors, including tensile strength and flexibility, are also enhanced, providing more robust and durable packaging solutions. Furthermore, nanocomposites enable tailored functionalities, such as altered surface wettability and increased hydrophobicity, further optimizing the protective and functional capabilities of the packaging system [71].

A wide range of organic and inorganic nanomaterials have been utilized in active packaging to achieve these benefits. For instance, nanocellulose and chitosan nanoparticles have been employed to enhance the physicochemical properties of polymeric films, while also demonstrating promising antimicrobial activity. These organic nanomaterials provide dual functionality by improving both the structural integrity and the active protection of food products [72].

Besides improving physical properties, inorganic nanoparticles, such as silver nanoparticles (AgNPs), are widely used as antimicrobial agents in food packaging due to their strong biocidal properties. Similarly, metal oxide nanoparticles, including zinc oxide (ZnO NPs) and titanium dioxide (TiO_2_ NPs), are commonly used in active packaging systems to generate reactive oxygen species, effectively eliminating microorganisms and enhancing food preservation [73].

By leveraging nanotechnology, active packaging systems can offer improved performance and functionality, aligning with the growing demand for safer and more sustainable food preservation methods.

## 4. Poly(lactic acid) (PLA) in Active Packaging

### 4.1. Poly(lactic acid) (PLA) Properties

Traditional polymers originating from petrochemicals already possess the requirements for a good packaging material; however, the long-term environmental impact of these materials has weakened their reputation, creating a demand for alternatives that can fulfill the same role while being biodegradable. These alternatives are commonly referred to as “green polymers”. Therefore, commercial and academic interest in utilizing biodegradable materials for food packaging has increased in recent years [74].

**Figure 7:**
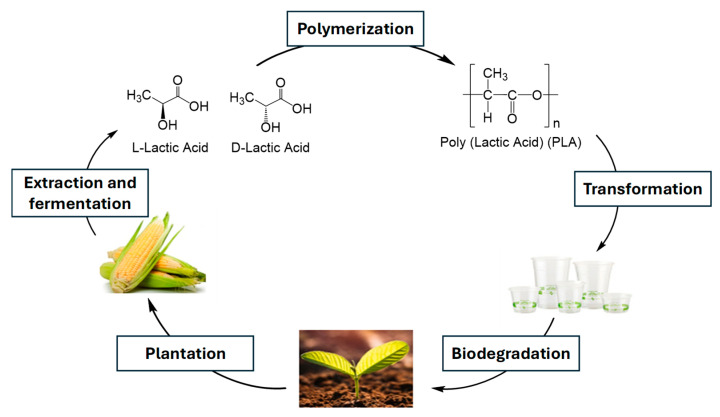
Life cycle of PLA (cultivation, fermentation and lactic acid polymerization, PLA transformation and composting).

Poly(lactic acid) (PLA) is proposed as an alternative solution, because of its attractive “green technology”, encompassing biocompatibility, renewability, and good thermal processability (e.g., by extrusion, film casting, and fiber spinning); moreover, the use of PLA in food packaging has already received commercial attention, e.g. in containers for water, juice, and yogurt, due to its appealing functional characteristics (high transparency, strong sealability, and excellent oil and grease resistance) [75,76]. In Figure 7, the PLA cycle is schematized.

On the other hand, PLA has several drawbacks: low heat resistance, making it unsuitable for hot liquids or applications with heat exposure; brittleness and limited mechanical strength; poor compatibility with recycling systems, as it can interfere with the conventional process; and weaker barrier properties for oxygen and water vapor compared to conventional plastics, largely due to its sensitivity to moisture.

Within this scenario, implementing some properties of PLA by introducing new AP functionalities may represent a breakthrough strategy.

### 4.2. Polylactic Acid as an Active Packaging Material

The use of PLA in active packaging systems has attracted significant interest. Researchers are exploring ways to maximize PLA’s advantageous properties as a packaging material while addressing its limitations, with promising outcomes [77]. In general, by incorporating nanoscale fillers, stiffness, tensile strength, and impact resistance of PLA are noticeably improved. Diverse nanoparticles may indeed create a more robust composite structure, often by forming strong interfacial interactions with the PLA matrix, thus helping to distribute stress more evenly and reduce brittleness. Additionally, nanoreinforcements can enhance the crystallinity of PLA, which further contributes to its mechanical strength. However, the extent of these improvements depends on factors such as filler type, dispersion quality and loading level. Proper dispersion and low filler content are crucial, as excessive loading can lead to agglomeration phenomena, reducing the overall effectiveness [78].

As discussed in the following sections, studies on PLA for active packaging primarily focus on two key areas: antimicrobial and antioxidant activity.

#### 4.2.1. Antimicrobial Activity

Fungal and bacterial contamination are major contributors to food spoilage, significantly impacting consumer appeal and resulting in great losses for the food industry. As a result, antibacterial packaging plays a crucial role in preserving product quality and extending shelf life.

PLA possesses properties that are highly compatible with various antimicrobial agents (see Table 1) and it has been widely investigated for controlling both pathogenic and spoilage microorganisms in biomedical and food applications [79]. The use of PLA in antimicrobial applications has been a popular topic of research in recent years. For example, Benhacine et al. [80] created an antimicrobial blend using PLA-PCL-silver exchanged montmorillonite (PCL/Ag–MMT, 3% wt) through a melt mixing process. The interaction between silver–MMT and PLA-PCL was assessed through ATR-FTIR tests. DSC tests showed an increase in PCL crystallinity compared to PLA crystallinity, indicating that silver–MMT particles were favorably dispersed in PCL. The filled blends showed enhanced thermal and mechanical properties, inhibiting pathogenic bacteria growth, demonstrating effective antibacterial activity.

Employing innovative electrospinning and soaking methods, Min et al. [81] engineered a novel humidity-responsive material. This material, a blend of porous PLA nano-fibers, thyme essential oil (TEO), PVA, and PEG, was designed to enhance its hydrophilicity through a PEG-PVA coating, as confirmed by water contact angle measurements. The inclusion of TEO endowed the material with potent antibacterial activity against *E. coli* and *S. aureus*, achieving over 99% inhibition. The release behavior in vitro was effectively regulated by adjusting the humidity. 

Building on advancements in antibacterial packaging, Javaherzadeh et al. [82] created PLA-based active packaging films that include nanochitosan (NCH) and *polylophiuminvolucartum* essential oil (PEI). When used to wrap chicken meat stored for ten days, the films proved effective, with results indicating that the packaged meat maintained greater freshness and displayed significantly lower bacterial activity compared to reference products. Further enhancing the antibacterial properties of PLA films, Wang et al. [83] used a coating method to prepare nisin-loaded chitosan–PLA films using PLLA (poly (L-lactic acid)). The results showed that when PLA was added to CS, hydrogen bonds were established, causing a cross-linking that decreased elongation at break. Nisin diffusion from the matrix to the solid culture medium decreased *S. aureus* growth. Similarly, Concilio et al. [84] added active azo compounds to PLA films via solvent casting and compounding methods to improve their antibacterial properties. The films showed excellent antibacterial activity against *S. aureus* and *C. albiceus* bacteria, with a total inhibition even at 0.1% concentration of the azo compound. DMA, TGA, DSC, and X-Ray characterizations revealed that the presence of azo compounds had no notable impact on the mechanical, thermal, and structural properties.

Suwanamornlert et al. [85] combined PLA with thymol and poly(butylene-succinate-co-adipate) (PBSA) to create antibacterial films. The mechanical properties showed good flexibility, with increased elongation at break and decreased Tg and Tf. In vitro trials showed promising results in inhibiting *Aspergillus* spp. and *Penicillum* spp. fungi. Additionally, 3% thymol films limited the growth of almost 20% of fungi, while 6% thymol films inhibited around 80% of them. The films were also used as packaging materials for bread, with a nine-day longer shelf life compared to PLA and commercial biaxially oriented polypropylene (BOPP) films, as determined by yeast and mold growth.

Yaowen et al. [86] loaded cinnamon essential oil (CEO) into chitosan (CS) via emulsion and ionic gelation methods and added the compound to PLA using the electrospinning method. SEM showed that the resulting composite (PLA-CS-CEO) had good interactions, which was also confirmed by the FTIR results. Water contact angle tests showed that the hydrophobicity of PLA decreased with the addition of CS-CEO (from 114° of pure PLA to 76.5°). The antibacterial tests showed good inhibition efficiency against *E. coli* and *S. aureus*.

Yan-Wu et al. [87] prepared films from a blend of PLA-PCL and thymol based on a solvent casting method. The stress—strain curve and differential scanning calorimetry (DSC) tests showed that the addition of thymol led to a decrease in the glass transition temperature (Tg) and a great increase in elongation at break, which means that thymol acted as a plasticizer and greatly improved film flexibility. The trials for antibacterial properties were carried out to test the inhibition of *S. aureus*, *Staphylococcus epidermidis*, and *E. coli*, and films with >9% thymol had the best inhibitory effects.

Sonseca et al. [88] made a blend of PLA–nanosilver–chitosan by melt compounding in a twin-screw extruder. The DSC results showed that a good miscibility was achieved, as also supported by the SEM and FTIR results. The test for mechanical properties showed an enhancement in toughness and elongation at break, rendering the material slightly more flexible. The antibacterial properties of the material were notable for its ability to inhibit Gram-negative and Gram-positive *S. aureus* bacteria. This effect was explained by the authors as a “dual effect” resulting from the antibacterial property of silver and the cationic character of chitosan.

Ahmed et al. [89] created composite films from PLA-PEG-CEO-Cu-Ag nanoparticles using a compression molding technique. The FTIR results indicate that the interaction between the CEO and the matrix was a chemical interaction, while the interaction with the nanoparticles was physical. From the thermal properties (DSC test), the results showed that the addition of nanoparticles increased the Tg by approximately 6 °C, but the addition of CEO decreased it by 24 °C. The films were tested against *L. monocytogenes*, *S. Typhimurium*, and *C. jejuni* bacteria inoculated in chicken meat for 21 days, and the trials showed great inhibitory activity, especially in the case of the films loaded with 50% CEO.

Spiridon et al. [90] blended grape waste and celery fibers with PLA as matrix. The mechanical properties did not improve much since the addition of grape waste led to a decrease in flexibility, and the worst case was with grape seeds. DTG tests showed that the overall degradation temperatures decreased, which means that the thermal stability was negatively affected. It was noted that the contact angle was lower than that of PLA, leading to an improvement in hydrophilicity. Antibacterial activity trials showed good inhibition results against the Gram-positive bacteria *S. aureus* (55.5%) and *E. coli* (20.6%).

Busolo et al. [91] conducted a thorough assessment of PLA biocomposites with high transparency, which were produced using solvent casting and contained a new silver-based antimicrobial layered silicate additive. DSC characterization showed that the melt temperature (T_m_) and the glass transition temperature (T_g_) remained unchanged with increasing nanoparticle content. Water vapor tests revealed an enhanced water barrier in the biocomposites, resulting in a potent antimicrobial effect. The trials showed that the silver-based nanoclays strongly inhibited the tested bacteria.

Torres-Giner et al. [92] incorporated zein hybrid fibers and thymol into PLA by electrospinning. This resulted in excellent mechanical properties. Tensile tests indicated that zein hybrid fibers have the potential to serve as reinforcing fillers, enhancing the mechanical properties of bioplastic PLA matrices and making them convenient for load-bearing applications. In contrast, gas barrier tests revealed that incorporating zein hybrid fibers as inner layers can be utilized to create multilayer packaging structures with improved gas barrier properties and aid in regulating the release of thymol. Antibacterial tests against *Listeria monocytogenes* CECT 5672 revealed that the notable reduction in bacterial growth was due to the controlled release of thymol.

Kara et al. [93] prepared submicron-sized PLA fibers encapsulating allyl isothiocyanate (AITC) and grafted them onto the surface of PLA films by electrospinning. SEM confirmed the merging of the two phases to form a bilayered fiber-grafted film. Tensile tests showed that the fiber-grafted PLA films (PfA-*g*-film) retained the mechanical properties of PLA. During the antibacterial trials that were conducted on *Listeria innocua* and *E. coli* K12, it was verified that the PLA-*g* films significantly inhibited their growth.

Ligaj et al. [94] prepared PLA films containing nanoparticles of zero valent iron (ZIV) via a casting method for dairy product packaging. The antimicrobial activity of the films was tested, and the results showed that PLA films with the addition of 3% ZVI (*w*/*w*) exhibited total bacterial inhibition. The use of goat cheese to verify the overall potential of the active packaging materials confirmed that the use of the ZVI/PLA coating on the polyolefin film extended the shelf life of the cheese up to 6 weeks.

**Table 1:** Collection of PLA-based antibacterial active packaging, showing the positive (↑) or negative (↓) effects on thermal, mechanical and antibacterial behavior.

Biomatrix	Active Compound	Effect on Thermal/Mechanical Properties	Active Agent Effect	Ref.
PLA/PCL	Silver-modified montmorillonite	-↑ Thermal stability -↑ Tensile results	-↑ Release duration to 30 days -Complete inhibition of bacteria growth	[80]
PLA/PEG/PVA	Thyme essential oil (TEO)	-↑ Hydrophilicity of fibers -↑ Effect in humid environment	-↑ Shelf life of strawberries: from 3 days to more than 5 days	[81]
PLA/Chitosan	Polylophium involucratum essential oil (PIE)	-No physical study carried out	-↑ Shelf life of chicken in refrigerated storage for more than 10 days	[82]
PLLA/Chitosan	Nisin	-With CS/PLA ratio 1:1 (*w*/*w*): -↑ tensile strength, ↓ elongation at break -With CS/PLA ratio below 1:1: -↓ tensile strength, ↓ elongation at break	The films showed antimicrobial activity against *S. aureus*	[83]
PLA	Azo compounds	Films retained the properties of the pure PLA	Complete inhibition of *S. aureus* biofilm formation even at 0.01% in PLA	[84]
PLA/PBSA	Thymol	-↓ T_g_ and T_f_ -↓ Tensile strength and Young’s modulus -↑ Elongation at break	Inhibited mold growth on bread from 6 to 9 days	[85]
PLA/Chitosan	Cinnamon essential Oil	-↑ Hydrophilic behavior	Good *E. coli* and *S. aureus* inhibition	[86]
PLA/PCL	Thymol	-Thymol: ↑ flexibility, ↓ crystallinity of PLA phase -PCL: ↓ Tg	Good *E. coli* and *L. monocytogenes* inhibition	[87]
PLA/Chitosan	Silver nanoparticles	-↑ Toughness and elongation at break	Good bacterial growth inhibition	[88]
PLA/PEG/PCL	ZnO/clove essential Oil (CEO)	-↓ Dynamic moduli by accelerating the polymer degradation -CEO acts as an efficient plasticizer	Good bacterial inhibition in scrambled eggs, at 4 °C for 21 days	[89]
PLA	Grape seeds	-3% of the filler does not affect mechanical properties -↓ Thermal stability	Grape seeds presented the highest antimicrobial activities	[90]
PLA	Silver-based nanoclay (MMT)	Crystallinity increased slightly and T_g_ increased with nanofiller loading	Strong antimicrobial activity	[91]
PLA/Zein/ Nanoclay	Thymol	-↑ 22% Young’s modulus -↑ 8% tensile strength at yield -↓ 31% elongation at break	Efficient bacterial growth inhibition	[92]
PLA	Alyll isothiocyanate	-↓ Young’s modulus and elongation at break	Good bacterial growth inhibition	[93]
PLA	Zero-valent iron (ZVI)	-Significant change in appearance and optical properties	3% of ZVI provided full inhibition of microbial growth	[94]

#### 4.2.2. Antioxidant Activity

Oxidation is one of the most significant factors impacting the shelf life of food. To address this challenge, incorporating oxygen scavengers presents numerous benefits, including the inhibition of microbial growth, prevention of rancidity (particularly in lipid-containing foods), suppression of oxidation, and preservation of color [95]. These advantages, together with the desirable properties of PLA, have heightened interest in the integration of antioxidant agents into PLA for food packaging applications.

Jamshidian et al. [96] investigated the release rates of various antioxidants from PLA to enhance food safety by reducing the direct addition of antioxidants. They measured the release of ascorbyl palmitate, α-tocopherol, and synthetic phenolic antioxidants (butylated hydroxyanisole (BHA), butylated hydroxytoluene (BHT), propyl gallate (PG), and tert-butylhydroquinone (TBHQ)) into ethanol solutions with different concentrations at 20 and 40 °C over 60 days, using high-performance liquid chromatography. The study examined diffusion (D) and partition (K) coefficients, revealing that PLA can effectively help in controlling food oxidation by serving as an antioxidant carrier. The release rate is influenced by the antioxidant’s molecular weight, Log P value, simulant polarity, and temperature. Catechin and epicatechin were also incorporated into PLA by Iñiguez-Franco et al. [97] on a pilot plant scale. The release rate of the antioxidants was evaluated, and their activity was assessed using the DPPH scavenging method. A correlation was established between the concentration of antioxidants and the percentage of DPPH radical scavenging for both catechin and epicatechin.

Relevant examples of antioxidant PLA composites are collected in Table 2 and discussed as follows. 

Mariño-Cortegoso et al. [98] examined if antioxidant compounds sourced from lemon and tomato by-products could be used as active compounds in different polymeric matrices, including PLA. Both extracts lead to a substantial improvement in water barrier properties of PLA, while PLA containing lemon extract at 4% was selected as the most suitable to extend the shelf-life of fat foods.

Papadopoulou et al. [99] prepared PLA/cocoa bean shells (CBSs) via a solution casting method and found from DSC results that the inclusion of CBS improved the crystallinity of PLA films and increased the physical properties of the composites. This was further confirmed by tensile test measurements, which showed an 80% increase in Young’s modulus with the inclusion of 75 wt% CBS while maintaining plasticity. An evaluation of the barrier properties, such as water vapor and oxygen permeability, showed favorable results with dependence on CBS content. The antioxidant activity was confirmed using the DPPH method. Moreover, incorporating CBS into PLA improved its biodegradation in aquatic environments, reaching 70% maximum biodegradability within 30 days.

Furthermore, Almasi et al. [100] prepared PLA films with TBHQ (3% wt) and modified cellulose nanofiber (MCNF) (8% wt), finding that MCNF incorporation significantly decreased the TBHQ release rate in a 95% ethanol simulant. DPPH tests showed that PLA-TBHQ films had better antioxidant activity than PLA-MCNF-TBHQ, although the latter retained its properties longer. Arroyo et al. [101] developed a 2D framework nanoparticle with antioxidant activity and added it to PLA-ATBC (acetyl tributyl citrate), creating a sustainable non-migratory system for active packaging. The material had good optical properties, with tensile and DSC tests confirming that ATBC enhanced film flexibility and decreased T_g_. The DPPH method indicated good antioxidant activity. Jamshidian et al. [102] formulated several PLA–phenolic antioxidant blends in a pilot plant extruder and conducted a comparative study on antioxidant ability, showing that temperature, ethanol concentration, polarity, Log P, and molecular volume affected the release rate, with PG exhibiting the best release rate.

Arrieta et al. [103] investigated the incorporation of catechin into a plasticized PLA–PHB blend, supplemented with ATBC for flexibility. A FESEM analysis confirmed effective dispersion, while the TGA, DSC, and tensile tests showed that catechin improved thermal stability and material rigidity. PHB presence increased PLA crystallinity, and the antioxidant activity showed significant efficacy in food preservation.

Lopusiewicz et al. [104] successfully integrated fungal melanin into PLA composites. The composites showed decreased strength at higher melanin concentrations, but minimal impact on glass transition temperature. Antioxidant activity increased with higher melanin concentrations, reaching 23%, contrasting with pure PLA’s negligible activity. The films also showed low antibacterial activity against *Enterococcus faecalis, Pseudomonas aeruginosa* and *Pseudomonas putida*.

Ramos et al. [105] incorporated thymol and modified montmorillonite into PLA for active packaging preparation. TGA and DSC analyses revealed minimal effects on PLA’s thermal stability and T_g_, while mechanical testing indicated reduced elastic modulus due to thymol’s plasticizing effect. The DPPH assay confirmed high encapsulation efficiency of thymol post-processing, resulting in significant antioxidant activity of the composite.

Di Maio et al. [106] examined the addition of α-tocopherol (α-TCP) as a natural antioxidant to PLA through cast extrusion blending. They found good dispersion but physical interactions between PLA and α-TCP. The latter had a plasticizing effect on PLA, increasing its break elongation by 95% and decreasing its glass transition temperature. The oxygen-scavenging capacity and absorption rate of PLA and PLA-TCP films were evaluated, revealing good antioxidant activity.

Ortenzi et al. [107] introduced some phenolic antioxidants during L-lactic acid polymerization to obtain antioxidant-active PLA films. The products were analyzed in terms of molecular weight (size exclusion chromatography) and thermal properties (DSC) and were compared with commercial-grade PLA used to produce flexible films. DPPH in vitro tests revealed that among the synthesized samples, PLA initiated with vanillyl alcohol displayed the best antioxidant properties. The thermal and molecular weight properties of PLA VA were found to be similar to those of commercial PLA, confirming its industrial viability. PLA-VA films were also tested in vivo, where they were used as a packaging material for salami. The films effectively improved the color stability and reduced lipid oxidation in salami during refrigerated storage.

Herskovitz et al. [108] employed reactive extrusion to graft metal chelating nitrilotriacetic acid (NTA) ligands onto PLA, resulting in the production of metal chelating PLA (PLA-g-NTA). The ATR-FTIR data confirmed that radical grafting on PLA indeed occurred, which led to a decrease in the crystallization and melting temperatures, as was also supported by the results of the DSC tests. Furthermore, PLA-g-NTA films exhibited notable radical scavenging and metal-chelating activities, as well as the ability to delay ascorbic acid degradation, highlighting their antioxidant potential.

De Dicastillo et al. [109] incorporated merken (an indigenous Chilean spice) as an antioxidant at 3% and 5% wt. percentages into PLA using the extrusion method. The thermal properties analysis indicated that adding merken reduced the thermal stability, crystallinity, and melting temperature compared to pure PLA. Mechanical tests, such as Young’s modulus and elongation at break, showed that the modified PLA was more flexible but less resistant. The release study of these antioxidants performed using the ABTS method followed Fick’s law.

**Table 2:** Collection of PLA-based antioxidant packaging, showing the positive (↑) or negative (↓) effects on thermal, mechanical and antioxidant behavior.

Biomatrix	Active Compound	Physicochemical Effects	Active Agent Effect	Ref.
PLA	Lemon/Tomato extracts	- ↑ water barrier properties of PLA	With lemon extract at 4%: ↑ shelf-life of fat foods.	[98]
PLA	Cocoa bean shells (CBSs)	-↑ 80% Young’s modulus -↑ 70% biodegradability	↑ Radical scavenging	[99]
PLA/ATBC	2D covalent organic framework COF	-Transparency maintained -↓ T_f_ -↑ 50% Elastic modulus	Slight ↑ in antioxidant activity	[100]
PLA/PHB	Catechin/ATBC	-Catechin: ↑ thermal stability -ATBC incorporation: ↓ elastic modulus and hardness	Performances maintained for more than 10 days in tested harsh environment	[103]
PLA	Melanin	-↓ Mechanical and properties -↑ Crystallinity	Good antioxidant activity	[104]
PLA	Thymol/MMT	Thymol addition: ↓ Tg of PLA, ↓ Elastic modulus, and ↑ elongation at break	Good antioxidant activity	[105]
PLA	α-tocopherol	-Did not change mechanical properties much	Good oxygen scavenging activity	[106]
PLA	Metal chelating: nitrilotriacetic acid (NTA)	↓ Contact angle values (increased hydrophilicity)	Good oxygen scavenging activity	[108]
PLA	Merken	-↓ Thermal stability -↓ Young’s modulus and elongation at break	Good radical scavenging activity	[109]

#### 4.2.3. Antibacterial–Antioxidant Activity

One significant upgrade of active packaging (AP) relies on the ability to incorporate multiple functional properties into a single packaging material. This multifunctionality has led researchers to explore, for example, the integration of both antibacterial and antioxidant agents with synergistic properties into polylactic acid (PLA). Mariana Ramos et al. [110] developed PLA films containing thymol and silver nanoparticles (Ag NPs) and assessed their antibacterial and antioxidant activities. Fourier transform infrared (FTIR) and X-ray techniques confirmed the successful blending of thymol and Ag NPs within the PLA matrix. Antioxidant activity, evaluated using the DPPH method over 15 days in ethanol, showed an increase in radical scavenging activity over time. The antibacterial tests demonstrated substantial efficacy against both Gram-negative *E. coli* RB and Gram-positive *S. aureus*, with a notably higher effectiveness against the latter.

Additionally, Burgos et al. [111] developed active films comprising PLA, polyhydroxybutyrate (PHB), and lactic acid oligomers (OLA) as plasticizers and carvacrol as active agent. The antioxidant efficacy of these PLA–PHB-based films was assessed using the DPPH radical method, revealing enhanced antioxidant and antimicrobial properties due to carvacrol. The presence of carvacrol exhibited greater antimicrobial effectiveness against *S. aureus* compared to *E. coli* over both short and extended incubation periods.

These studies underscore the potential of incorporating antibacterial and antioxidant agents into polymer matrices to develop multifunctional active packaging materials with superior performance. Extensive research on antioxidant and antibacterial PLA films and coatings has already led to the commercialization of some products. While the simultaneous incorporation of multiple active agents may compromise certain overall properties, PLA-based AP materials remain a promising alternative to traditional synthetic polymers. They exemplify the potential of biocomposites to enhance food safety and quality while paving the way for continued advancements in eco-friendly packaging solutions.

#### 4.2.4. PLA Engineering for Higher AP Efficiency

Recent changes in consumer demand emphasize fresh, packaged foods like meat, vegetables, and fruits. Consequently, a primary challenge for the food packaging industry is preserving food freshness throughout packaging and storage. This has driven efforts to develop effective packaging materials that address concerns such as cost, food compatibility, consumer appeal, and multifunctional properties—including resistance to water vapor and oil, mechanical strength, heat resistance, antioxidant, and antibacterial activity. Leveraging new methodologies, processing technologies, and advanced materials is key to optimizing packaging performance [112]. In particular, engineering the chemical–physical properties of PLA to enhance its effectiveness in AP applications has been under consideration recently.

One advance is the development of PLA-based polymeric foams, which are lightweight and provide excellent thermal insulation, cushioning, and protection for food products. These foams can be used for active packaging applications, since they can act as a carrier for active agents (e.g., antimicrobial, antioxidant, or moisture absorbers) [113].

The intrinsic slow crystallization ability of PLA may also restrict its extensive applications. Controlling the crystallinity of PLA is an emerging area of research, particularly for regulating the release of bioactive compounds. Crystallinity affects the material’s diffusion properties: a higher degree of crystallinity usually results in slower release due to the restricted movement of active compounds through the polymer matrix. Moreover, studies have shown that gas permeability depends on chain orientation, amorphous phase morphology, lamellar arrangement, and crystalline forms of PLA. Therefore, achieving good gas permeability in PLA-based packaging presents a challenge in aligning the PLA lamellae along the gas diffusion path, requiring the application of proper crystallization protocols to create a more efficient structure of PLA [114]. An increased crystallinity also improves the thermal stability of PLA-based packaging, making it more resistant to higher temperatures, which is valuable for certain food processing or packaging environments [115].

As far as processing is concerned, 3D printing allows for tailored packaging designs that can be adapted to specific product needs. Complex geometries, customized packaging features, and precise distribution of active agents (e.g., antimicrobial or antioxidant compounds) within the structure are possible through this emerging technique [116].

Supercritical fluid technology was also applied in combination with cocrystallization engineering, to develop novel eugenol (EU)-loaded PLA nanocomposite foams. Eugenol-phenazine (EU-PHE) cocrystals, produced through a solvent-free mechanochemical method, were incorporated into PLA foams using supercritical solvent impregnation. The study explored the impact of cocrystallization and the incorporation of Cloisite30B^®^ (C30B nanoclay) on the release kinetics of EU, revealing a slower, prolonged release and enhanced antimicrobial activity. The developed materials effectively inhibited the attachment of *L. monocytogenes* and *S. Enteritidis* [117].

Multilayering is another highly effective strategy for creating active packaging [118]. This technique involves the combination of different materials or layers within a single package, each serving a specific function, leading to enhanced performance. For example, an outer layer could provide mechanical strength and resistance to external factors; a middle layer could embed active agents (antioxidants, antimicrobials, or moisture absorbers) that slowly release over time to protect the food, and an inner layer in direct contact with the food may be designed for the controlled release of bioactive compounds. As a matter of fact, a multifunctional polylactic acid (PLA)-based bioplastic with antifouling and antibacterial properties was prepared by using a dual-coating approach. The surface, designed with micro/nano-scale roughness and low surface energy, incorporated polyhexamethylene guanidine hydrochloride (PHMG) as a bactericidal agent. The film exhibited superhydrophobicity (water contact angle of 154.3°) and strong antibacterial performance against *E. coli* and *S. aureus*. It was also useful to inhibit biofilm formation, extending the shelf life of fresh chicken breast by up to eight days [119].

## 5. Conclusions

Active packaging offers significant potential, not only by enhancing product protection and shielding against environmental and human impacts but also by contributing to waste reduction in the food sector. By extending the shelf life of perishable items and reducing spoilage, active packaging helps minimize food waste at various stages of the supply chain, from production to retail and consumer use. Combining active packaging with smart functionalities will offer many possibilities to provide real-time condition monitoring, giving valuable information to both consumers and retailers regarding product freshness and safety during transport and storage [120]. This approach aims to achieve two main objectives: first, to monitor the condition of packaged food by providing real-time information about its quality during transportation and storage, and second, to respond dynamically to changes in the food or its environment, enabling an automated system for continuous food quality and safety monitoring and remediation.

However, further research is needed to address existing preservation challenges and bridge gaps in current technology. To scale up biocomposite production, several critical issues must be tackled, including poor mechanical performance, limited processability, and high production costs—factors that contribute to extended development timelines before market launch [121]. In addition, the mechanical properties of plant fibers, which vary depending on their origin, present additional obstacles to their effective use in biocomposites. These challenges are compounded by the irregular geometry, surface roughness, and hydrophilic nature of plant fibers, which often lead to poor adhesion with hydrophobic polymer matrices. This incompatibility can result in reduced mechanical strength, limiting their performance in demanding applications. Additionally, variability in the quality and composition of biomass fillers due to factors such as climate, harvest methods, and storage conditions poses significant challenges for consistent production. Ensuring a reliable supply of raw materials is further complicated by logistical issues, such as seasonal availability, high transportation costs, and the need for extensive preprocessing to meet manufacturing requirements. Overcoming these obstacles is critical to unlocking the full potential of biocomposites and ensuring their sustainable integration into various industries [122].

Despite the challenges, biocomposite materials hold enormous potential for diverse applications and could eventually replace synthetic materials entirely. Biodegradable materials are especially critical in addressing environmental pollution caused by micro- and nanoplastics, particularly in regions grappling with uncontrolled landfilling. Successful examples include the use of agricultural by-products such as straw and husks [123] and forestry resources like wood fibers and lignin [124], which serve as valuable inputs for biocomposite production. Additionally, harnessing bioactive molecules, such as secondary metabolites, from plant and vegetable waste streams offers exciting opportunities for innovation. Compounds such as polyphenols, flavonoids, terpenoids, and essential oils, extracted from agricultural and food processing residues, provide natural antimicrobial and antioxidant properties [125]. These bioactive agents contribute to food preservation by reducing microbial growth and oxidative spoilage while supporting circular economy goals through waste valorization. Marine waste, including algae and shells, is also under exploration for its exceptional properties, such as high strength-to-weight ratios and biodegradability, making it an ideal resource for sustainable biocomposite production [126].

On the other hand, the development of biobased composite materials may introduce additional challenges, especially concerning the unintended release of molecules and nanoparticles, primarily into food systems and subsequently into the environment. To address these issues, it is essential to carefully optimize compatibilization strategies between the matrix and the filler. While chemical treatments and processes can mitigate these limitations, toxicological assessments and end-of-waste studies must be implemented to ensure the safety of biocomposites throughout their lifecycle, evaluating the effects of adding nanomaterials on the biodegradability of the polymer.

From a future perspective, an ideal biocomposite for active packaging should enable efficient, large-scale production without compromising ease of disposal. This means designing materials that balance functional durability with environmental sustainability, supporting both circular economy initiatives and the industry’s growing need for scalable, eco-friendly solutions.

Concerning PLA-based derivatives, novel designs are necessary to achieve various goals, such as antimicrobial activity, oxygen scavenging, and moisture control, without compromising their already limited mechanical properties, barrier capabilities, and biodegradability. Combining PLA with different molecules, nanofillers, or natural extracts may create smart packaging with targeted properties to further improve preservation for specific product categories. Additionally, blending PLA with other biopolymers or functional layers, such as polyhydroxyalkanoates (PHA), starch, or cellulose, can synergistically enhance mechanical, barrier, and degradation properties while maintaining a sustainable profile. Optimizing techniques, including simple surface modification strategies, copolymerization with multifunctional cross-linkers, crystallinity control, foaming, multi-layering, and encapsulation, are expected to be promising avenues for advancing PLA-based food packaging systems.

## Data Availability

No new data were created or analyzed in this study.

## Abbreviations

AP	Active Packaging
CH	Chitosan
CR	Regenerated cellulose films
DMA	Dynamic mechanical analysis
DPPH	2,2-diphenyl-1-picrylhydrazyl
DSC	Differential scanning calorimetry
EC	European Commission
FTIR	Fourier transform infrared spectroscopy
LNP	Lignin nanoparticles
MMT	Montmorillonite
NTA	Nitrilotriacetic acid
PBSA	Poly(butylene succinate)
PBTA	Poly(butylene adipate terephthalate)
PCL	Poly(caprolactone)
PE	Poly(ethylene)
PHAs	Poly(hydroxyalkanoate)
PHB	Poly(hydroxybutyrate)
PLA	Poly(lactic acid)
PP	Poly(propylene)
PS	Poly(styrene)
PVA	Poly(vinyl alcohol)
SCPC	Starch containing polymer compounds
SEM	Scanning electron microscopy
TGA	Thermogravimetric analysis
TTI	Time temperature indicators
